# Supplementary material for: Neighbourhood property value and type 2 diabetes mellitus in the Maastricht study: A multilevel study
Source: PLoS One. 2020 Jun 8;15(6):e0234324. doi: 10.1371/journal.pone.0234324 (PMC7279598; doi:10.1371/journal.pone.0234324)
Supplement: S3 Table — N = 2,056. Random slope for household income (covariance unstructured). (DOCX) [file pone.0234324.s003.docx]

| **Supplemental table 1.3:** Multilevel logistic regression of T2DM (0=no, 1=yes). N=2,056.  Random slope for household income (covariance unstructured). | | | | | | | | | | | | | | |
| --- | --- | --- | --- | --- | --- | --- | --- | --- | --- | --- | --- | --- | --- | --- |
|  | | **Model 1** | | | | **Model 2** | | | | | | **Model 3** | | |
|  | | AIC: 2231.84  VPC: 9.2% | | | | AIC: 2030.71  VPC: 4.9% | | | | | | AIC: 2018.50  VPC: 3.2% | | |
|  | | **Odds Ratio** | **95% C.I.** | | | **Odds Ratio** | | **95% C.I.** | | | | **Odds Ratio** | **95% C.I.** | |
| **Intercept** | | 0.31 | [0.25, 0.37] | | | 0.06 | | [0.02, 0.16] | | | | 0.05 | [0.02, 0.14] | |
| **Age** | |  |  | |  | 1.05 | | [1.04, 1.07] | | | | 1.05 | [1.04, 1.07] | |
| **Sex** | |  |  | |  |  | |  | | |  |  |  |  |
| Male | |  |  | |  | 1.00 | | - | | | | 1.00 | - | |
| Female | |  |  | |  | 0.31 | | [0.24, 0.39] | | | | 0.31 | [0.25, 0.40] | |
| **Educational Level** | |  |  | |  | 0.50 | | [0.28, 0.87] | | | | 0.53 | [0.30, 0.93] | |
| **Occupational Status** | |  |  | |  | 0.51 | | [0.28, 0.91] | | | | 0.53 | [0.30, 0.95] | |
| **Household Income** | |  |  | |  | 0.52 | | [0.20, 1.32] | | | | 0.69 | [0.27, 1.75] | |
|  | |  |  | |  |  | |  | | |  |  |  |  |
| **Property Value** | |  |  | |  |  | |  | | |  |  |  |  |
| Extremely high | |  |  | |  |  | |  | | |  | 1.00 | - | |
| Moderately high | |  |  | |  |  | |  | | |  | 1.14 | [0.76, 1.71] | |
| Moderately low | |  |  | |  |  | |  | | |  | 1.27 | [0.85, 1.90] | |
| Extremely low | |  |  | |  |  | |  | | |  | 2.38 | [1.58, 3.56] | |
|  |  | | |  | | |  | |  |  |  |  |  |  |
| **Random part*** | **Estimates** | | | **Standard Error** | | |  | |  |  |  |  |  |  |
| Variance: income | 0.02 | | | 0.17 | | |  | |  |  |  |  |  |  |
| Variance: intercept | 0.09 | | | 0.12 | | |  | |  |  |  |  |  |  |
| Covariance | -0.04 | | | 0.23 | | |  | |  |  |  |  |  |  |

* *Random coefficients are relative to model 3 only*
