# Supplementary material for: Neighbourhood property value and type 2 diabetes mellitus in the Maastricht study: A multilevel study
Source: PLoS One. 2020 Jun 8;15(6):e0234324. doi: 10.1371/journal.pone.0234324 (PMC7279598; doi:10.1371/journal.pone.0234324)
Supplement: S7 Table — N = 2,056. (DOCX) [file pone.0234324.s007.docx]

| **Supplemental table 2.** Multilevel logistic regression of T2DM (0=no, 1=yes) with 4-digits postal code and neighbourhood SES. N=2,056. | | | | | | | | | |
| --- | --- | --- | --- | --- | --- | --- | --- | --- | --- |
|  | **Model 1** | | | **Model 2** | | | **Model 3** | | |
|  | AIC: 2236.02  VPC: 7.3% | | | AIC: 2030.72  VPC: 4.1% | | | AIC: 2027.02  VPC: 2.1% | | |
|  | **Odds Ratio** | **95% C.I.** | | **Odds Ratio** | **95% C.I.** | | **Odds Ratio** | **95% C.I.** | |
| **Intercept** | 0.31 | [0.25, 0.39] | | 0.07 | [0.02, 0.17] | | 0.06 | [0.02, 0.17] | |
| **Age** |  |  |  | 1.05 | [1.04, 1.07] | | 1.05 | [1.04, 1.07] | |
| **Sex** |  |  |  |  |  |  |  |  |  |
| Male |  |  |  | 1.00 | - | | 1.00 | - | |
| Female |  |  |  | 0.31 | [0.24, 0.39] | | 0.31 | [0.25, 0.39] | |
| **Educational Level** |  |  |  | 0.50 | [0.28, 0.81] | | 0.51 | [0.89, 0.90] | |
| **Occupational Status** |  |  |  | 0.50 | [0.28, 0.89] | | 0.50 | [0.28, 0.89] | |
| **Household Income** |  |  |  | 0.49 | [0.19, 1.23] | | 0.52 | [0.21, 1.30] | |
|  |  |  |  |  |  |  |  |  |  |
| **Neighbourhood SES** |  |  |  |  |  |  |  |  |  |
| Extremely high |  |  |  |  |  |  | 1.00 | - | |
| Moderately high |  |  |  |  |  |  | 1.35 | [0.87, 2.10] | |
| Moderately low |  |  |  |  |  |  | 1.58 | [0.99, 2.53] | |
| Extremely low |  |  |  |  |  |  | 1.99 | [1.30, 3.05] | |

*Data for this analysis came from the Netherlands Institute of Social Research, which provides SES scores for each 4-digits postal code area in the Netherlands for specific years. The SES score is based on mean income, percentage of low incomes, percentage of low educated residents and percentage of unemployed residents in each area.*

The Netherlands Institute of Social Research. Sociaal en cultureel planbureau statusscores [In Dutch]. <https://www.scp.nl/Onderzoek/Lopend_onderzoek/A_Z_alle_lopende_onderzoeken/Statusscores.> Date last accessed 5 Jan 2017.

Knol F. Statusontwikkeling van wijken in Nederland 1998-2010 [In Dutch]. <https://www.scp.nl/Publicaties/Alle_publicaties/Publicaties_2012/Statusontwikkeling_van_wijken_in_Nederland_1998_2010.> Date last update: November, 2012.
