# Supplementary material for: Neighbourhood property value and type 2 diabetes mellitus in the Maastricht study: A multilevel study
Source: PLoS One. 2020 Jun 8;15(6):e0234324. doi: 10.1371/journal.pone.0234324 (PMC7279598; doi:10.1371/journal.pone.0234324)
Supplement: S8 Table — N = 2,077. (DOCX) [file pone.0234324.s008.docx]

| **Supplemental table 3.1:** Multilevel linear regression of fasting plasma glucose tolerance status. N=2,077. | | | | | | | | | |
| --- | --- | --- | --- | --- | --- | --- | --- | --- | --- |
|  | **Model 1** | | | **Model 2** | | | **Model 3** | | |
|  | AIC: 7976.18  VPC: 4.4% | | | AIC: 7815.84  VPC: 3.5% | | | AIC: 7799.16  VPC: 1.8% | | |
|  | **Coeff.** | **95% C.I.** | | **Coeff.** | **95% C.I.** | | **Coeff.** | **95% C.I.** | |
| **Intercept** | 5.97* | [5.85, 6.09] | | 5.42* | [4.84, 5.99] | | 5.17* | [4.56, 5.78] | |
| **Age** |  |  |  | 0.02* | [0.01, 0.03] | | 0.02* | [0.01, 0.03] | |
| **Sex** |  |  |  |  |  |  |  |  |  |
| Male |  |  |  | 0.00 | - | | 0.00 | - | |
| Female |  |  |  | -0.76* | [-0.89, -0.62] | | -0.74* | [-0.88, -0.60] | |
| **Educational Level** |  |  |  | -0.23 | [-0.58, 0.12] | | -0.17 | [-0.53, 0.18] | |
| **Occupational Status** |  |  |  | -0.40* | [-0.77, -0.26] | | -0.37* | [-0.74, -0.00] | |
| **Household Income** |  |  |  | -0.19 | [-0.74, 0.65] | | -0.03 | [-0.58, 0.51] | |
|  |  |  |  |  |  |  |  |  |  |
| **Property Value** |  |  |  |  |  |  |  |  |  |
| Extremely high |  |  |  |  |  |  | 0.00 | - | |
| Moderately high |  |  |  |  |  |  | 0.00 | [-0.25, 0.26] | |
| Moderately low |  |  |  |  |  |  | 0.06 | [-0.19, 0.32] | |
| Extremely low |  |  |  |  |  |  | 0.59* | [0.32, 0.85] | |

* Statistically significant (P<0.005)
