# Supplementary material for: Neighbourhood property value and type 2 diabetes mellitus in the Maastricht study: A multilevel study
Source: PLoS One. 2020 Jun 8;15(6):e0234324. doi: 10.1371/journal.pone.0234324 (PMC7279598; doi:10.1371/journal.pone.0234324)
Supplement: S9 Table — N = 1,942. (DOCX) [file pone.0234324.s009.docx]

| **Supplemental table 3.2:** Multilevel linear regression of 2-h plasma glucose tolerance status. N=1,942 | | | | | | | | | |
| --- | --- | --- | --- | --- | --- | --- | --- | --- | --- |
|  | **Model 1** | | | **Model 2** | | | **Model 3** | | |
|  | AIC: 10855.46  VPC: 5.4% | | | AIC: 10675.48  VPC: 3.2% | | | AIC: 10656.84  VPC: 1.7% | | |
|  | **Coeff.** | **95% C.I.** | | **Coeff.** | **95% C.I.** | | **Coeff.** | **95% C.I.** | |
| **Intercept** | 7.63* | [7.33, 7.94] | | 3.76* | [2.35, 5.19] | | 2.99* | [1.50, 4.48] | |
| **Age** |  |  |  | 0.10* | [0.08, 0.12] | | 0.10* | [0.08, 0.12] | |
| **Sex** |  |  |  |  |  |  |  |  |  |
| Male |  |  |  | 0.00 | - | | 0.00 | - | |
| Female |  |  |  | -1.23* | [-1.57, -0.89] | | -1.20* | [-1.54, -0.86] | |
| **Educational Level** |  |  |  | -1.60* | [-2.47, -0.72] | | -1.47* | [-2.35, -0.60] | |
| **Occupational Status** |  |  |  | -0.15 | [-0.07, 0.76] | | -0.08 | [-0.99, 0.84] | |
| **Household Income** |  |  |  | -0.89 | [-2.21, 0.43] | | -0.48 | [-1.80, 0.85] | |
|  |  |  |  |  |  |  |  |  |  |
| **Property Value** |  |  |  |  |  |  |  |  |  |
| Extremely high |  |  |  |  |  |  | 0.00 | - | |
| Moderately high |  |  |  |  |  |  | 0.10 | [-0.51, 0.71] | |
| Moderately low |  |  |  |  |  |  | 0.39 | [-0.23, 1.00] | |
| Extremely low |  |  |  |  |  |  | 1.54* | [0.89, 2.18] | |

* Statistically significant (P<0.005)
