# Supplementary material for: Neighbourhood property value and type 2 diabetes mellitus in the Maastricht study: A multilevel study
Source: PLoS One. 2020 Jun 8;15(6):e0234324. doi: 10.1371/journal.pone.0234324 (PMC7279598; doi:10.1371/journal.pone.0234324)
Supplement: S10 Table — (DOCX) [file pone.0234324.s010.docx]

| **Supplemental table 4:** Multilevel logistic regression of comparing a) normal glucose levels individual with prediabetes (N=1,562); b) normal glucose levels individual with T2DM (N=1,739); c) prediabetes with T2DM (N=811). | | | | | | |  | |
| --- | --- | --- | --- | --- | --- | --- | --- | --- |
|  | **a) Prediabetes  vs**  **Normal glucose level (model 3)** | | | **b) T2DM vs Normal glucose level (model 3)** | | | **c) Prediabetes vs T2DM (model 3)** | |
|  | AIC: 1531.30  VPC: 0.0% | | | AIC: 1782.94  VPC: 2.8% | | | AIC: 1045.30 VPC: 0.0% | |
|  | **Odds Ratio** | **95% C.I.** | | **Odds Ratio** | **95% C.I.** | | **Odds Ratio** | **95% C.I.** |
| **Intercept** | 0.18 | [0.01, 5.06] | | 0.03 | [0.01, 0.08] | | 1.36 | [0.35, 5.36] |
| **Age** | 1.06 | [1.04, 1.07] | | 1.06 | [1.05, 1.08] | | 1.01 | [0.99, 1.03] |
| **Sex** |  |  |  |  |  |  |  |  |
| Male | 1.00 | - | | 1.00 | - | | 1 | - |
| Female | 0.71 | [0.55, 0.91] | | 0.29 | [0.22, 0.37] | | 0.41 | [0.30, 0.56] |
| **Educational Level** | 0.92 | [0.48, 1.78] | | 0.51 | [0.28, 0.92] | | 0.55 | [0.25, 1.19] |
| **Occupational Status** | 0.80 | [0.40, 1.62] | | 0.51 | [0.28, 0.92] | | 0.58 | [0.27, 1.27] |
| **Household Income** | 0.46 | [0.17, 1.29] | | 0.58 | [0.22, 1.54] | | 1.33 | [0.38, 4.71] |
|  |  |  |  |  |  |  |  |  |
| **Property Value** |  |  |  |  |  |  |  |  |
| Extremely high | 1.00 | - | | 1.00 | - | | 1 | - |
| Moderately high | 0.88 | [0.62, 1.26] | | 1.13 | [0.73, 1.74] | | 1.24 | [0.80, 1.93] |
| Moderately low | 0.95 | [0.66, 1.37] | | 1.24 | [0.81, 1.92] | | 1.38 | [0.88, 2.16] |
| Extremely low | 1.22 | [0.84, 1.76] | | 2.62 | [1.69, 4.07] | | 1.84 | [1.19, 2.84] |
