# Supplementary material for: Neighbourhood property value and type 2 diabetes mellitus in the Maastricht study: A multilevel study
Source: PLoS One. 2020 Jun 8;15(6):e0234324. doi: 10.1371/journal.pone.0234324 (PMC7279598; doi:10.1371/journal.pone.0234324)
Supplement: S11 Table — N = 1,908. Only neighbourhoods with at least 10 respondents included*. (DOCX) [file pone.0234324.s011.docx]

| **Supplemental table 5:** Multilevel logistic regression of T2DM (0=no, 1=yes). N=1,908.  Only neighbourhoods with at least 10 respondents included*. | | | | | | | | | |
| --- | --- | --- | --- | --- | --- | --- | --- | --- | --- |
|  | **Model 1** | | | **Model 2** | | | **Model 3** | | |
|  | AIC: 2048.88  VPC: 9.7% | | | AIC: 1877.30  VPC: 5.5% | | | AIC: 1862.24  VPC: 2.2% | | |
|  | **Odds Ratio** | **95% C.I.** | | **Odds Ratio** | **95% C.I.** | | **Odds Ratio** | **95% C.I.** | |
| **Intercept** | 0.29 | [0.23, 0.36] | | 0.06 | [0.02, 0.17] | | 0.03 | [0.01, 0.10] | |
| **Age** |  |  |  | 1.04 | [1.03, 1.06] | | 1.05 | [1.03, 1.06] | |
| **Sex** |  |  |  |  |  |  |  |  |  |
| Male |  |  |  | 1.00 | - | | 1.00 | - | |
| Female |  |  |  | 0.31 | [0.24, 0.39] | | 0.32 | [0.25, 0.41] | |
| **Educational Level** |  |  |  | 0.46 | [0.26, 0.83] | | 0.48 | [0.27, 0.87] | |
| **Occupational Status** |  |  |  | 0.59 | [0.32, 1.08] | | 0.63 | [0.34, 1.16] | |
| **Household Income** |  |  |  | 0.62 | [0.23, 1.65] | | 0.84 | [0.32, 2.22] | |
|  |  |  |  |  |  |  |  |  |  |
| **Property Value** |  |  |  |  |  |  |  |  |  |
| Extremely high |  |  |  |  |  |  | 1.00 | - | |
| Moderately high |  |  |  |  |  |  | 1.29 | [0.84, 2.01] | |
| Moderately low |  |  |  |  |  |  | 1.37 | [0.89, 2.11] | |
| Extremely low |  |  |  |  |  |  | 2.66 | [1.72, 4.12] | |

* *Number of neighbourhoods= 48; observation per neighbourhood: min 11, avg 40, max 135.*
