# Supplementary material for: Neighbourhood property value and type 2 diabetes mellitus in the Maastricht study: A multilevel study
Source: PLoS One. 2020 Jun 8;15(6):e0234324. doi: 10.1371/journal.pone.0234324 (PMC7279598; doi:10.1371/journal.pone.0234324)
Supplement: S12 Table — N = 2,056. Property value reverse coded. (DOCX) [file pone.0234324.s012.docx]

| **Supplemental table 6:** Multilevel logistic regression of T2DM (0=no, 1=yes). N=2,056. Property value reverse coded. | | | | | | | | | |
| --- | --- | --- | --- | --- | --- | --- | --- | --- | --- |
|  | **Model 1** | | | **Model 2** | | | **Model 3** | | |
|  | AIC: 2231.84  VPC: 9.2% | | | AIC: 2030.71  VPC: 4.9% | | | AIC: 2016.54  VPC: 2.2% | | |
|  | **Odds Ratio** | **95% C.I.** | | **Odds Ratio** | **95% C.I.** | | **Odds Ratio** | **95% C.I.** | |
| **Intercept** | 0.31 | [0.25, 0.37] | | 0.06 | [0.02, 0.16] | | 0.04 | [0.01, 0.11] | |
| **Age** |  |  |  | 1.05 | [1.04, 1.07] | | 1.05 | [1.04, 1.07] | |
| **Sex** |  |  |  |  |  |  |  |  |  |
| Male |  |  |  | 1.00 | - | | 1.00 | - | |
| Female |  |  |  | 0.31 | [0.24, 0.39] | | 0.31 | [0.25, 0.40] | |
| **Educational Level** |  |  |  | 0.50 | [0.28, 0.87] | | 0.53 | [0.30, 0.93] | |
| **Occupational Status** |  |  |  | 0.51 | [0.28, 0.91] | | 0.53 | [0.29, 0.95] | |
| **Household Income** |  |  |  | 0.52 | [0.20, 1.32] | | 0.69 | [0.27, 1.75] | |
|  |  |  |  |  |  |  |  |  |  |
| **Property Value** |  |  |  |  |  |  |  |  |  |
| Extremely low |  |  |  |  |  |  | 1.00 | - | |
| Moderately low |  |  |  |  |  |  | 0.53 | [0.36, 0.79] | |
| Moderately high |  |  |  |  |  |  | 0.48 | [0.32, 0.72] | |
| Extremely high |  |  |  |  |  |  | 0.42 | [0.28, 0.63] | |
