# Supplementary material for: Neighbourhood property value and type 2 diabetes mellitus in the Maastricht study: A multilevel study
Source: PLoS One. 2020 Jun 8;15(6):e0234324. doi: 10.1371/journal.pone.0234324 (PMC7279598; doi:10.1371/journal.pone.0234324)
Supplement: S13 Table — (DOCX) [file pone.0234324.s013.docx]

| **Supplemental table 7:** Percentage distribution of the variables before excluding cases with missing data. | | |
| --- | --- | --- |
|  | **Total** | **T2DM** |
|  | **percentage** | **percentage** |
| **Sex** |  |  |
| Female | 48.5 | 67.7 |
| Male | 51.5 | 32.3 |
|  |  |  |
| **Age** |  |  |
| 40 - 53 | 23.0 | 13.5 |
| 54 - 59 | 22.1 | 18.6 |
| 60 - 65 | 27.6 | 26.9 |
| 66 -75 | 27.3 | 41 |
|  |  |  |
| **Educational Level** |  |  |
| University education | 4.5 | 9.7 |
| Higher professional education | 21.3 | 28.6 |
| Intermediate vocational, Higher secondary | 27.8 | 28.2 |
| Primary, lower general/vocational | 46.4 | 33.5 |
|  |  |  |
| **Occupational status (ISEI08 score)** |  |  |
| 88.9 - 70.6 | 17.7 | 23.7 |
| 70.5 - 56.1 | 23.0 | 25.8 |
| 56.0 - 39.1 | 24.0 | 25.6 |
| 39.0 - 13.2 | 35.3 | 24.9 |
|  |  |  |
| **Household Income (€)** |  |  |
| 6,000 - 2,437 | 39.5 | 30.1 |
| 2,386 - 1,888 | 21.0 | 20.3 |
| 1,875 - 1,509 | 22.9 | 25.9 |
| 1,502 - 424 | 16.6 | 23.7 |
| **Property Value (€)** |  |  |
| 581,000 - 262,000 | 24.6 | 15.5 |
| 261,000 - 226,000 | 25.2 | 21.5 |
| 225,000 - 169,000 | 23.9 | 25.0 |
| 168,000 - 125,000 | 26.3 | 38.0 |
