# Supplementary material for: Neighbourhood property value and type 2 diabetes mellitus in the Maastricht study: A multilevel study
Source: PLoS One. 2020 Jun 8;15(6):e0234324. doi: 10.1371/journal.pone.0234324 (PMC7279598; doi:10.1371/journal.pone.0234324)
Supplement: Attachment — Submitted filename: Response to Reviewers.docx [file pone.0234324.s014.docx]

**Rebuttal Letter**

**Reviewer #1:** This paper essentially builds on the previously published analyses of the cross-sectional relationship between socioeconomic status and diabetes prevalence in the baseline data collection for the Maastrict Cohort Study. Another recent publication (included as reference 2) explores in much more detail the association with a wide range of individual level markers of socioeconomic status. This analysis uses the potential to link the dataset to neighbourhood property values to extend the use of this dataset to explore the association with this area-level variable.

RESPONSE: As the reviewer highlights, this present study has some commonalities with a previously published one (Qi *et al.*, 2019), relying on the same dataset. However, the specific focus of interest is radically different between the two, whereby the latter has the primary objective of inquiring which dimension of SES or material deprivation (the only additional predictor with respect to our study) is more prominently associated with the onset of the disease. Differently, in our study, individual SES variables were included to examine the net effect of a contextual variable independently of individual characteristics; we thus needed an overall measurement of individual SES, without needing to go deep in assessing which specific socioeconomic characteristic is mostly associated with T2DM onset.

Whilst the approach to analysis and presentation of findings seems appropriate and clear, both the rationale for choosing property value as a proxy of environmental determinants of diabetes risk and the caveats in terms of assuming this is a causal association in the interpretation of the findings could be more adequately addressed as suggested below:

1. As property value reflects both an element related to the size and quality of individual properties and an element related to the perceived attractiveness of the area (e.g. local amenities, access to public transport, quality of local green spaces etc) it is difficult without any information on, or adjustment for, the former to know the extent to which property value reflect area rather than property specific characteristics. This makes it difficult to be confident of the authors assertion that property value is measuring area SES rather being a proxy for individual variable not adjusted for.

RESPONSE: We surely agree with the fact that property value encompasses both the aspects mentioned. However, the use of this indicator as a proxy of area-level SES is not novel in the field of public health, as some studies cited in our manuscript (but there are others) demonstrate (Coffee *et al.*, 2013; Drewnowski *et al.*, 2007). Specifically, Coffee and colleagues report on the usefulness of relying on residential property as an area-SES measure, also referring to the empirical literature (e.g. – but not only – Bourassa *et al.*, 2003: “Do housing submarkets really matter?”) that assessed the contribution of different housing characteristics in determining housing price, which seems to converge on the idea that location is by far the most relevant one. Accordingly, we could safely state that if taken as a single value (i.e. the value of each unique property), it would be difficult to discern to which features (e.g. number of rooms, dimension, energetic class, location, etc.) the variable is mostly influenced by, but once the values are averaged by area, the variable confidently acts as an indicator of area attractiveness, being this last the prominent dimension in determining housing prices, according to the “hedonic pricing” literature. By stringently controlling for the individual SES measures, that might indeed be proxies for the personal property characteristics, we sought to quantify the area/contextual effect. Indeed, when we performed the sensitivity analysis with a different area-SES variable (area-level education, unemployment, income), the results reached were close to the one from the main analysis with property value, suggesting that this last acts as a reliable area-SES indicator. Given the relevance of the issue highlighted, we added a brief passage in the text clarifying this (lines 87-91 in the marked-up document).

2. It appears from the previously published analysis that there may be other confounding variables and residual confounding by individual factors (for which property value may be a proxy) cannot be excluded as explanation for findings.

RESPONSE: We agree with the reviewer that we cannot assume that our models are fully specified: there are certainly other confounding factors that if included may alter to some extent the findings reached. This issue is present also in the published analyses to which the reviewer refers. For instance, information on individual susceptibility to T2DM (“biology” or “genetic”) is hardly available, and consequently seldom included in models aimed at evaluating T2DM risk. However, in our opinion this does not undermine all the studies that do not include this measurement, especially if the main focus is different. On the other hand, adding too many variables representing the same concept may lead to the risk of overfitting, explaining a greater extent of variability without a counterbalance in data quality and models estimation. That is the reason why we pursued a more parsimonious strategy, including covariates in the models exclusively if we had specific hypotheses related to their influence on the outcome and are related to the exposure, and not just because they were available. The most important confounder is probably income, and we controlled for it and – more stringently – for two other individual SES measures. We added a passage in the discussion mentioning this issue (lines 367-376).
Moreover – and also related to the previous point – the several robustness checks we performed with different variables’ operationalizations and units were conceived to deal with some of the issues raised, that is to reach higher confidence that the findings reached were not achieved by chance.

3. The lack of a relationship with prediabetes is also a finding that suggests it is less likely that this cross-sectional association is due to areas characteristics associated with property values being on the causal pathway to diabetes. Authors need to at least discuss the potential explanation of reverse causality with health status (including obesity and diabetes) being on a causal pathway that results in an individual living in an area with lower property values.

RESPONSE: We believe that the lack of relationship with prediabetes is driven by the small amount of data when segmenting the variable into three categories, but we have no means to demonstrate this at present. Also, the fact that a gradient is present in each comparison suggests that it could be just a question of small numbers, rather than absolute lack of relationship. It would be interesting to test this conjecture with the additional cohort study data, when available. Though this is undoubtedly ambiguous, on the other hand the linear models with continuous variables (FPGT and 2-h PGT) as outcome still show the presence of a territorial gradient in T2DM. (Added: lines 338-341)
We added a statement also concerning reverse-causality (Added: lines 357-359)

**Reviewer #2:** Overall comment

An interesting study with ‘daring’ interpretation that perhaps require further data and explanation.

Background

Further information and examples of the different environmental and material aspects of the different categories of property in particular between that of extreme high and extreme low would aid understanding of this variable on T2DM. Further comparisons to other countries and discussion has been quite well done under the Discussion section but probably requiring a more attention to the differences between the two extreme categories, as shown by the result.

RESPONSE: We took account of all the comments and issues raised, and we hope that our answers address the overall concerns about the interpretation of our findings.

Lines 67-70: the statement is less accurate as the review reported that most of the included studies that adjusted for lifestyle behaviours and psychosocial factors had insignificant effects except one study solely in women.

RESPONSE: True, we corrected the passage in the text (lines 69-70).

Methods

1. Slightly more information on “extensive phenotyping approach” may help understanding of the Maastricht Study.

RESPONSE: We added a more detailed statement in the text (lines 112-114). Further information on the study and the data are available from the article cited, where the *The Maastricht Study* is presented (Schram *et al.*, 2014).

2. Disclose also the differences between the included and excluded respondents from the aspects of NPV based on the CBS Statistics Netherlands. If this information was lost (n=76), please explain how did this happen because isn’t it a readily available data?

RESPONSE: Some respondents had missing data on neighbourhood property value simply because it was not possible to geo-reference them in any of the neighbourhood of the study area. The field containing the address of residence in the Maastricht Study (which have been linked to the CBS), may have been filled erroneously or left blank by some respondents, with the consequent loss of information. For instance, when implementing the matching, the address of some respondents resulted to be located outside the municipalities involved in the study, probably because of postcodes reported incorrectly. Other addresses were instead missing. This is a typical issue when dealing with self-reported questionnaires, and unfortunately there is little we could do dealt with it. Differences in NVP – resulted to be irrelevant – have been added in the paragraph (lines 135-136).

3. Were all patients in the regional Diabetes Patient Registry selected and invited? How were the oversampling of people with T2DM executed and completed?

RESPONSE: Yes. More in detail, as explained in the article presenting *The Maastricht Study (Schram et al.*, 2014; p. 4):

*Participants are recruited from mass media campaigns and from the municipal registries (Gemeentelijke Basis Administratie; GBA) and the regional Diabetes Patient Registry (see below) via mailings. The representation with the source population in the study region is monitored continuously (see Fig. 3) and is aligned with postal codes. The regional Diabetes Patient Registry is kept by the regional association of General Practitioners (Zorg in Ontwikkeling (ZIO)) and the Maastricht University Medical Centre. This registry includes individuals that apply to their GP with health complaints which lead to the diagnosis of T2DM, and individuals that are diagnosed with T2DM after cardiovascular screening. The registry virtually includes all T2DM individuals in primary, secondary or tertiary medical care in the ‘‘Maastricht and Heuvelland’’ region.*

The oversampling of type 2 diabetes was primarily through recruitment via the regional Diabetes Registry but some participants with type 2 diabetes also came from the recruitment of the general population.

4. How good is the regional Diabetes Patient Registry in terms of coverage of the people with a diagnosis of T2DM in the Southern part of the Netherlands? Could you provide a comparison between those included respondents and those in the registry at large from the aspects of NPV (if this is possible)? Supplemental Table 7 shows that the extreme groups of the occupational status and property value quartiles could be over- and under-represented.

RESPONSE: The introduction of bundled payment for diabetes care in 2007 in the Netherlands led to the origination of care groups in primary care in the Netherlands (e.g. see Mohnen *et al.* (2017) Cost Variation in Diabetes Care across Dutch Care Groups?). These regional care groups are led by general practitioners (GPs) who receive extra reimbursement (and personnel) for each patient with type 2 diabetes they treat. Negotiations with the health care insurance companies is performed by the regional care organisation, that – amongst others – also monitors quality of care parameters and runs the diabetes electronic record file in each GP practise, which is also the basis of the regional Diabetes Patient Registry. As there are no costs for the patient when treated by the GP in the Netherlands, the barriers to care are very low. The Maastricht Study was performed within and in close collaboration with the regional care organisation. Please note that around 6% of the total type diabetes population in the region is treated in secondary care. Therefore, we assume that more than 90% of the patients diagnosed with type 2 diabetes are covered by this system.
Unfortunately, we don’t have information to compare general characteristics of type 2 diabetes patients in our study to those in the Registry.

5. I believe the municipal registries hold the total residential information. Could you provide a comparison between those included respondents and those in the regions at large from the aspects of NPV (if this is possible)?

RESPONSE: We can only compare educational level between our sample and the whole Southern Limburg Population. However, this is very relevant, since it, if anything, shows that the Maastricht Study underestimates the percent of diabetes in lower educated and overestimates the percent in the higher educated. This does not fit with a bias resulting in overestimating the health inequalities in the Maastricht Study. The sampling in the diabetes register and in the general population register was random and the findings below are strongly suggestive of no differential (i.e. stronger) sampling of patients from poor areas.

|  | GGD/RIVM/CBS 40-64 y (2012) – Zuid-Limburg (n = 758 van de 11000)  % | DMS 40-75 y (2010 en later)  (n= 949 van de 3400)  % |
| --- | --- | --- |
| Geen opleiding  Lager onderwijs  LBO  MAVO  MBO  HAVO/VWO  HBO  Universiteit  Rest/overig | 5.7  9.4  26.9  15.0  19.8  6.3  13.1  3.2  0.7 | 1.1  7.1  19.3  18.1  21.5  6.1  21.2  4.4  1.2 |

*Table: Distribution of educational level within diabetics: Southern Limburg vs The Maastricht Study (source: Gezondheidsmonitor 2012).*

6. Line 158: please provide more detail on the normalization procedure.

RESPONSE: The three indicators were rescaled in order to vary between 0 and 1, with the formula $\frac{X-X min}{X\max- X min}$ , for two reasons. First, the variables were necessarily reported in different units of measurement, and consequently their coefficients in the models fitted (especially the linear ones) were not directly comparable between them if not normalized or standardized. Second, the software used (STATA 15) reported errors when fitting the random intercept and slope models with the individual SES variables in the original scales, whilst rescaling makes the data better processable. The normalization procedure allowed fixing this issue.

Results

Suggest to put the total (n=…) on Table 1 caption itself and for the T2DM column.

RESPONSE: Done.

Providing key reason/s for the 3 sensitivity analyses would be appreciated here although are quite well discussed in the Discussion. Is the third one for a support of (small) sample size?

RESPONSE: We added in the text a short explanation of the rationale of each additional analysis (lines 260-262; 265-266; 268-269).
The third sensitivity analysis (same models as the main one, but including only neighbourhoods with at least 10 respondents) was performed to address the issue of neighbourhoods with few respondents. This is an extra check, given that multilevel models apply a so-called “shrinkage factor”, which tends to pull the coefficients deriving from units with low numerosity towards the mean (the overall intercept), while conversely leaving unaltered the coefficients deriving from units with higher numbers. This a sort of weighting based on sample numerosity of each area-level unit, not of the overall sample. Accordingly, when applying such techniques there is no bias if some units are less populated than others are, but it is a common and useful practice to perform an additional test to check if including these underrepresented areas may have altered the results. As expected, this is not the case in our analysis: the results were similar between the two models. For more details on the shrinkage factor, please check <http://www.bristol.ac.uk/cmm/learning/videos/residuals.html>.

Discussion

Could NPV (and its personal SES indicators) a correlate to health behaviours that has an effect on T2DM? Isn’t it established that people of higher SES more available and capable for healthier lifestyles? Greener and more walkable spaces and healthier food environments will not benefit anyone who is not motivated to self-regulation and self-care, or unavailable and not affordable for them. The former (NPV) may act as the external motivator and the later (personal SES) may complement as the internal motivator and both would function through regular/consistent health behaviours on T2DM. The association could be bidirectional and inherited through generations (shared personal and social traits) in the majority. What would be a more plausible explanation for the association between NPV and T2DM?

RESPONSE: Yes, this may be the case. People of higher SES are better able to adopt healthy behaviours, but this is not attributable exclusively to motivation and explicit choices. Our perspective is less individualistic and more structuralist, in the way in which we are interested in studying which aspects of the material and psychosocial context of living are able to influence the onset of the disease. For instance, low and high SES individuals may be located in the same neighbourhood with no green/walkable spaces, but this could be a problem only for the firsts, given that those more equipped with material resources may find an easy way to cope with that (e.g. having a car to move to another area to exercise or having a budget to allocate for a gym subscription). Accordingly, for the lower SES individuals it is not a question of motivations, free action and choices, but of possibilities/opportunities, as influenced by the wider context in which people are embedded. We believe that there is surely some correlation between NPV, individual SES, behaviours/lifestyle, and the specific aim of this research is to disentangle this issue, assessing the “net” effect of NVP on T2DM, *ceteris paribus.* Moreover, as reported at the beginning of the section, the independence of NVP from individual SES implies that living in a disadvantage place is a risk factor itself, also for the better off. Accordingly, NVP and SES do not measure the same thing, as the numbers of low (/high) SES individuals living in high (/low) NVP areas demonstrate. We surely recognize the importance of the mechanisms highlighted by the reviewer, which we think are not excluded from our research. The current paper, however, has the purpose of finding a possible additional contextual effect. Moreover, we did not adjust for these variables since – as stated by the reviewer – these are likely mediators, and not confounders. Controlling for mediators would result in underestimated odds ratios for the socioeconomic factors.

Lines 311-316: do discuss a bit more on this statistical observation. The extremely low quartile property value seems to be substantially different from all the others (Supplemental Table 6). From another perspective, concluding and suggesting to achieve the external features of the moderately low property value is probably more achievable by many stakeholders to also enjoy its health benefits similar to that in the extremely high property value.

RESPONSE: We did not think about it, but this is very interesting. As the reviewer suggests, it seems that urban policies aimed at improving the quality of the living environment should not need to bring every neighbourhood at the highest level – it might also be unfeasible – and that just moving from a situation of extreme neighbourhood deprivation to one of relative improvement could be very useful. This does not intend to provide a justification for trivial interventions, but rather to highlight that the interventions we claim for are not utopian, and that risk exposure in the most deprived areas could be reduced with less efforts.
We added this consideration in the text (347-352).

It is probably good to remind the readers that the results are likely not/less applicable to lower/middle income countries.

RESPONSE: Done (lines 288-289).

Are ‘greener and more walkable spaces and healthier food environments’ true observed differences between the different NPV in the southern Netherlands? Are not these relatively accessible to almost all occupants?

RESPONSE: Unfortunately, we have no data to demonstrate this at hand, and it would require additional research that goes beyond the scope of our study. However, we found this specific research article examining the relationship between property value and the aforementioned characteristics in the Netherlands:

*Luttik, J. (2000). The value of trees, water and open space as reflected by house prices in the Netherlands. Landscape and urban planning, 48(3-4), 161-167.*

Though it is quite dated, it seems to suggest that the association to which we refer is real.
Despite this is the only specific study concerning the Dutch case, the international literature* seems to confirm this general trend in the urban environment, and we see no reason why Southern Limburg/the Netherlands should be an exception.

*See: *Wolch, J. R., Byrne, J., & Newell, J. P. (2014). Urban green space, public health, and environmental justice: The challenge of making cities ‘just green enough’. Landscape and urban planning, 125, 234-244.*

Accessibility to services is a function of distance, but also of material resources. As discussed above, lower SES people may be limited in their mobility which restrict their access to health-protecting/enhancing resources, so that we cannot easily assume that they have the potential to reach all the areas within the municipality (or the surrounding ones) where they live. On the contrary, those with less purchasing power are generally more prone to rely on services and amenities available in the proximity of their residence, whilst the better off have usually greater chance to move to reach desired facilities. Accordingly, if the issue of mobility has to be taken into account, it rather might widen the already existing socioeconomic inequalities in access to services. We added a statement about this issue (lines: 309-319)
